# Supplementary material for: Digital spatial profiling of segmental outflow regions in trabecular meshwork reveals a role for ADAM15
Source: PLoS One. 2024 Feb 23;19(2):e0298802. doi: 10.1371/journal.pone.0298802 (PMC10889904; doi:10.1371/journal.pone.0298802)
Supplement: S1 Table — (DOCX) [file pone.0298802.s001.docx]

**S1 Table of Log2FC values for each segment in high and low outflow regions of TM**. Values that were found to be outliers in whisker boxplots for *ADAM15, LDB3, CRKL*, and *BGN* are in red and indicated with an asterisk.

| **High Flow** | | | | | | | | | | | | | | |
| --- | --- | --- | --- | --- | --- | --- | --- | --- | --- | --- | --- | --- | --- | --- |
|  | **Donor 1 Segments** | | | | | **Donor 2 Segments** | | | | **Donor 3 Segments** | | | | |
|  | 1 | 2 | 3 | 4 | 5 | 1 | 2 | 3 | 4 | 1 | 2 | 3 | 4 |  |
| *ADAM15* | 6.3 | 5.8 | 6.2 | 6.3 | 6.1 | 6.7 | 5.3 | 6.5 | 5.9 | 6.9 | 6.9 | 7.0 | 7.1 |  |
| *LDB3* | 3.2 | 3.5 | 3.3 | 3.5 | 3.6 | 2.8 | 3.4 | 3.3 | 3.3 | **1.0*** | **0.5*** | 2.5 | 2.5 |  |
| *CRKL* | 3.6 | 4.0 | 2.8 | 2.9 | 3.7 | 2.6 | 3.3 | 3.6 | 3.7 | 1.5 | **-0.5*** | 2.3 | 2.7 |  |
| *BGN* | 2.7 | 2.0 | 1.5 | 2.8 | 3.0 | 1.9 | 3.7 | 1.5 | 1.9 | 0.5 | 0.5 | 2.0 | 1.1 |  |

| **Low Flow** | | | | | | | | | | |
| --- | --- | --- | --- | --- | --- | --- | --- | --- | --- | --- |
|  | **Donor 1 Segments** | | **Donor 2 Segments** | | | **Donor 3 Segments** | | | | |
|  | 1 | 2 | 1 | 2 | 3 | 1 | 2 | 3 | 4 | 5 |
| *ADAM15* | 5.3 | 5.3 | 5.2 | 5.1 | 5.2 | 5.7 | 6.0 | 6.2 | 5.9 | 6.6 |
| *LDB3* | 4.7 | 5.5 | 4.3 | 3.9 | 4.3 | 5.5 | 3.6 | 3.5 | 4.6 | 3.9 |
| *CRKL* | 4.3 | 5.5 | 4.4 | 4.3 | 4.1 | 5.4 | 3.5 | 3.0 | 4.9 | 4.2 |
| *BGN* | 3.9 | 3.9 | 3.7 | 2.8 | 3.3 | 4.6 | 3.0 | 2.2 | 3.4 | 2.2 |
