# Supplementary material for: Digital spatial profiling of segmental outflow regions in trabecular meshwork reveals a role for ADAM15
Source: PLoS One. 2024 Feb 23;19(2):e0298802. doi: 10.1371/journal.pone.0298802 (PMC10889904; doi:10.1371/journal.pone.0298802)
Supplement: S2 Table — (DOCX) [file pone.0298802.s002.docx]

**S2** **Table of Genes differentially expressed in high outflow regions of TM.** Unless otherwise noted with a citation, the function of genes were determined using GeneCards®, the human gene database.

| **Gene** | **Protein Name** | **Function** | **Log2**  **FC** | **Adj p value** |
| --- | --- | --- | --- | --- |
|  |  | **Cell Cycle** |  |  |
| *MZT2B* | Mitotic Spindle Organizing Protein 2B | Mitotic spindle formation | +1.1 | 0.004 |
| *CDC25C* | M-phase inducer phosphatase 3 | Phosphatase activates G2 cells into prophase; Inhibitor of apoptosis, | -1.0 | 0.01 |
| *CDK19* | Cyclin dependent kinase 19 | Ser/thr kinase, cell proliferation | -1.2 | 0.008 |
| *ZCCHC10* | zinc finger CCHC-type containing 10 | Role in apoptosis; stabilizes p53 protein [1] | -1.3 | 0.0001 |
| *HAUS5* | HAUS augmin like complex subunit 5 | Maintenance of microtubules during mitotic spindle formation | -1.4 | 0.03 |
| *BAK1* | Bcl-2 homologous antagonist/killer | Bcl2 antagonist; anti-apoptotic activity | -1.4 | 0.005 |
| *ZCCHC2* | Zinc Finger CCHC-Type Containing 2 | Inhibits polyubiquitination and activation of c-Myc.[2] | -1.4 | 0.03 |
| *FAM131C* | Family with Sequence Similarity 131 Member C) | Inhibitor of apoptosis | -1.5 | 0.01 |
| *PTMS* | Parathymosin | Inhibits senescence and NF-kB activity | -1.6 | 0.01 |
| *FAIM2* | Fas Apoptotic Inhibitory Molecule 2 | Inhibitor of apoptosis, activates Wnt pathway | -1.7 | 0.01 |
|  |  |  |  |  |
|  |  | Cytoskeleton |  |  |
| *MAPT* | Tau | Microtubule associated protein, transport of cargo along microtubule | 1.2 | 0.008 |
| *WIPF3* | WAS/WASL interacting protein family member 3 | Actin binding; regulates actin dynamics for phagocytic cup, endosomal recycling and rho GTPase signaling. Upregulated by corticosteroids? | -1.0 | 0.03 |
| *DIAPH2* | Diaphanous Related Formin 2 | Actin binding protein, member of formin family | -1.1 | 0.03 |
| *ANK1* | Ankyrin 1 | Attaches integral membrane proteins to cytoskeletal elements | -1.1 | 0.03 |
| *BMERB1* | BMERB domain containing 1 | Microtubule binding protein | -1.1 | 0.005 |
| *KRTAP5-8* | Keratin associated protein 5-8 | Increases rigidity of intermediate filaments | -1.2 | 0.02 |
| *LAD1* | Ladinin 1 | Binds filamin-bundled actin filaments; anchors filament to basement membrane [3, 66] | -1.5 | 0.01 |
| **Gene** | **Protein Name** | **Function** | **Log2**  **FC** | **Adj p value** |
|  |  | **Immune Response** |  |  |
| *ING4* | Inhibitor of growth protein 4 | Represses transcriptional activity of RelA/NF-κB | -1.2 | 0.03 |
| *KIR3DL1* | Killer cell immunoglobulin like receptor, 3 Ig domains and long cytoplasmic tail 1 | Activation of NK cells; signals via ITIM receptor | -1.3 | 0.01 |
| *BGN* | Biglycan | Wnt and TGFβ signaling, DAMP ligand for TLR4 | -1.4 | 0.03 |
| *PIANP* | PILR alpha associated neural protein, | Member of Ig superfamily; involved in immune regulation | -1.4 | 0.03 |
| *FAM131C* | Family with sequence similarity 131 member C | Inhibitor of apoptosis | -1.5 | 0.01 |
| *PTMS* | Parathymosin | Inhibits NF-kB; Restores BMP signaling | -1.6 | 0.006 |
|  |  |  |  |  |
|  |  | **Channels/Transporters** |  |  |
| *SUMO1* | Small Ubiquitin Like Modifier 1 | Nuclear transport, modulates gating properties of potassium channels | +1.4 | 0.02 |
| *KCTD1* | K2+ Channel Tetramerization Domain Containing 1 | Modulation of Wnt signaling; enhances ubiquitination & degradation of β-catenin | -1.0 | 0.04 |
| *KCNIP1* | K2+ Voltage-Gated Channel Interacting Protein 1 | K-channel that regulates A-type currents in response to Ca2+; hypertension | -1.0 | 0.04 |
| *KCTD21* | K2+ channel tetramerization domain containing 21 | Ubiquitination of HDAC1; regulates transcription factor GLI1. | -1.2 | 0.03 |
| *CACNG7* | Ca2+ voltage-gated channel auxiliary subunit gamma 7 | Regulates l-type Ca2+ channel | -1.3 | 0.04 |
| *CPNE5* | Copine 5 | Ca2+ binding protein with integrin like sequences; mediates Ca2+ dependent responses at cell membrane | -1.4 | 0.01 |
|  |  |  |  |  |
|  |  | **Intracellular transport** |  |  |
| *CHMP1B* | Charged multivesicular body 1B | Endosomal sorting | +1.0 | 0.04 |
| *STX1B* | Syntaxin 1b | Exocytosis | -1.0 | 0.04 |
| *MFSD3* | Major facilitator superfamily domain containing 3 | Involved in proton transmembrane transport | -1.3 | 0.01 |
| *VAMP1* | Vesicle associated membrane protein 1 | Docking and/or fusion of synaptic vesicle | -1.3 | 0.02 |
| *TBC1D13* | TBC1 domain family member 13 | GTPase activator towards RAB3; regulates vesicle transport and glut4 a glucose transporter | -1.4 | 0.01 |
| *SLC5A10* | Solute carrier family 5 member 10 | Transporter of glucose | -1.4 | 0.01 |
|  |  |  |  |  |
|  |  | **Translation or DNA Repair** |  |  |
| *EIF2A* | Eukaryotic Translation Initiation Factor 2A | Translation; Involved in ER stress | +1.2 | 0.04 |
| *TMA7* | Translation Machinery Associated 7 Homolog | Translation | +0.9 | 0.02 |
| *BRME1* | break repair meiotic recombinase recruitment factor 1 | DNA repair | -1.0 | 0.03 |
| *EME2* | Essential Meiotic Structure-Specific Endonuclease Subunit 2 | DNA repair | -1.0 | 0.05 |
| *POLD4* | DNA polymerase delta 4 | DNA polymerase | -1.3 | 0.01 |
|  |  |  |  |  |
|  |  | **Miscellaneous** |  |  |
| *WSB2* | WD Repeat and SOCS Box Containing 2 | Mediates ubiquitination and proteasomal degradation of target proteins. | +1.0 | 0.05 |
| *TMEM30A* | transmembrane protein 30A | Enables aminophospholipid flippase activity | +0.9 | 0.04 |
| *AMPD2* | Adenosine Monophosphate Deaminase 2 | Deamination of AMP to IMP | -0.9 | 0.006 |
| *GAL3ST4* | Galactose-3-O-Sulfotransferase 4 | Transfer of sulfate to beta-1,3-linked galactose residues in O-linked glycoproteins | -1.1 | 0.04 |
| *SULT1A4* | Sulfotransferase family 1A member 4 | catalyze the conjugation of sulfate to hormones, neurotransmitters etc; associated with UPR pathway | -1.2 | 0.05 |
| *HSD3B2* | Hydroxy-delta-5-steroid dehydrogenase | Biosynthesis of hormonal steroids | -1.2 | 0.02 |
| *LRRC75B* | Leucine rich repeat containing 75B | May modulate Erk1/2 signaling | -1.3 | 0.0001 |
| *HSD3B7* | Hydroxy-delta-5-steroid dehydrogenase | Bioynthesis of hormonal steroids and bile acids from cholesterol | -1.4 | 0.04 |
| *PRR32* | Proline rich 32 | uncharacterized | -1.5 | 0.04 |

References

1. Ning Y, Hui N, ZHuo Y, Sun W, Du Y, Liu S, et al. ZCCHC10 suppresses lung cancer progression and cisplatin resistance by attenuating MDM2-mediated p53 ubiquitination and degradation. Cell Death & Disease 2019;10:414.

2. Dai H, Yan M, Li Y. The zinc-finger protein ZCCHC2 suppresses retinoblastoma tumorigenesis by inhibiting HectH9-mediated K63-linked polyubiquitination and activation of c-Myc. Biochem Biophys Res Comm. 2020;521:533-8.

3. Chiasson-MacKenzie, McClatchey. EGFR-induced cytoskeletal changes drive complex cell behaviors: The tip of the iceberg. Sci Signal. 2018;11:9473.

4. Roth L, Srivastava S, Lindzen M, Sas-Chen A, Sheffer M, Lauriola M, et al. SILAC identifies LAD1 as a filamin-binding regulator of actin dynamics in response to EGF and a marker of aggressive breast tumors. Sci Signal. 2018;11:eaan0949.
