# Supplementary material for: Digital spatial profiling of segmental outflow regions in trabecular meshwork reveals a role for ADAM15
Source: PLoS One. 2024 Feb 23;19(2):e0298802. doi: 10.1371/journal.pone.0298802 (PMC10889904; doi:10.1371/journal.pone.0298802)
Supplement: S3 Table — (DOCX) [file pone.0298802.s003.docx]

**S3 Table of Log2FC values of ECM proteins and integrins for each segment in high and low outflow regions of TM**. Values that were found to be an outlier in whisker boxplots are indicated in red and marked with an asterisk. ** Expression levels of these genes were lower and values were determined by adjusting Q3 normalization using Filter 0.1% UDV =2 in the software program.

| **High Flow** | | | | | | | | | | | | | | |
| --- | --- | --- | --- | --- | --- | --- | --- | --- | --- | --- | --- | --- | --- | --- |
|  | **Donor 1 Segments** | | | | | **Donor 2 Segments** | | | | **Donor 3 Segments** | | | | |
|  | 1 | 2 | 3 | 4 | 5 | 1 | 2 | 3 | 4 | 1 | 2 | 3 | 4 |  |
| *FN1* | -0.1 | 0.8 | 1.0 | 1.0 | 1.0 | 2.0 | **-0.5*** | 0.5 | -0.9 | 0.5 | 1.0 | 1.0 | 1.1 |  |
| *DCN* | 0.9 | 2.9 | 0.5 | 1.5 | 0.7 | 1.2 | 0.5 | 0.5 | -0.9 | 2.3 | 1.5 | 2.8 | 1.1 |  |
| *SPARC* | 1.9 | 0.9 | 0.5 | 1.8 | 2.2 | 1.2 | 1.8 | 2.5 | -0.9 | 1.1 | 0.5 | 0.5 | 0.1 |  |
| *MMP2* | 3.6 | 1.5 | 0.5 | 2.6 | 3.3 | 0.8 | 0.5 | 0.5 | 0.7 | 2.5 | 0.5 | 2.1 | 0.1 |  |
| *VCAN* | 0.9 | 1.2 | -0.5 | 1.5 | 0.2 | 0.9 | -0.5 | 0.5 | 1.5 | 1.5 | 1.1 | 1.8 | 0.7 |  |
| *THBS2* | 1.5 | 0.8 | -0.5 | -0.5 | -0.3 | -0.1 | 1.0 | 3.3 | 2.1 | 1.8 | 1.5 | 0.5 | -0.9 |  |
| *TGFB2*** | 0.8 | 1.5 | 0.6 | 0.8 | 2.1 | 2.0 | 0.6 | 2.5 | 0.6 | 0.6 | 1.2 | 2.5 | 3.7 |  |
| *TIMP1* | 4.3 | 3.6 | 2.9 | 4.1 | 3.7 | 3.2 | 1.1 | 3.3 | 2.1 | 2.1 | 3.6 | 3.6 | 1.7 |  |
| *ITGA2*** | 0.8 | 2.0 | 1.2 | 1.6 | 2.1 | 2.5 | **3.1** | 1.2 | 3.7 | 0.6 | 3.1 | 1.2 | 2.5 |  |
| *ITGA4*** | 0.8 | 1.5 | 0.6 | 0.8 | 1.2 | 0.5 | 1.9 | 1.2 | 1.2 | 1.2 | 3.1 | 1.2 | 2.5 |  |
| *ITGA5* | 1.9 | 0.5 | 2.1 | 0.5 | 1.7 | 0.5 | -0.5 | 0.5 | 1.1 | -0.5 | 0.5 | 0.5 | 0.1 |  |
| *ITGA7*** | 1.6 | 0.5 | 1.2 | 3.3 | 0.4 | 3.0 | 0.6 | 2.5 | 1.9 | 3.7 | 1.2 | 0.6 | 2.5 |  |
| *ITGA8*** | 2.5 | 1.5 | 2.5 | 1.6 | 1.2 | 1.0 | 1.9 | 1.2 | 2.5 | 1.2 | 2.5 | 2.5 | 1.2 |  |
| *ITGA9*** | 0.8 | 1.0 | 1.2 | 2.5 | 0.4 | 2.0 | 0.6 | 2.5 | 0.6 | 1.2 | 0.6 | 0.6 | 1.2 |  |
| *ITGB1* | 2.9 | 1.2 | 1.8 | 2.5 | 2.7 | 1.9 | 1.1 | 0.5 | -0.9 | 2.6 | 1.5 | 2.5 | 1.1 |  |
| *ITGB3* | 0.9 | **-1.1*** | 1.1 | 1.8 | 1.5 | 0.5 | 1.5 | 0.5 | 1.5 | 0.5 | 1.5 | 0.5 | 1.7 |  |

| **Low Flow** | | | | | | | | | | |
| --- | --- | --- | --- | --- | --- | --- | --- | --- | --- | --- |
|  | **Donor 1 Segments** | | **Donor 2 Segments** | | | **Donor 3 Segments** | | | | |
|  | 1 | 2 | 1 | 2 | 3 | 1 | 2 | 3 | 4 | 5 |
| *FN1* | 0.7 | 1.7 | -1.3 | -0.9 | -0.5 | 0.5 | 0.5 | -0.1 | 1.0 | 1.2 |
| *DCN* | 0.1 | 0.5 | 0.7 | 0.1 | 0.5 | 0.5 | 0.5 | -0.1 | 0.5 | 1.2 |
| *SPARC* | 2.0 | -0.1 | 1.0 | 0.1 | 1.8 | 2.0 | 0.5 | 0.9 | 1.1 | 2.5 |
| *MMP2* | 1.7 | -1.1 | 2.1 | 2.3 | 0.5 | 1.5 | 0.5 | 0.9 | 1.9 | 2.3 |
| *VCAN* | 0.1 | 0.5 | 0.2 | 1.1 | 0.5 | 2.1 | 0.5 | 2.2 | 1.5 | 1.2 |
| *THBS2* | -0.9 | -1.1 | 0.2 | -0.9 | 0.5 | 0.5 | 2.1 | 1.9 | 1.1 | -1.2 |
| *TGFB2*** | 0.6 | 1.0 | 2.1 | 0.6 | 0.8 | 1.2 | 2.5 | 0.8 | 0.6 | 4.0 |
| *TIMP1* | 4.4 | 1.7 | 4.1 | 3.8 | 2.5 | 1.5 | 2.1 | 1.5 | 1.5 | 2.9 |
| *ITGA2*** | 1.2 | 1.5 | 1.2 | 2.5 | 3.3 | 1.2 | 2.5 | 0.8 | 1.9 | 3.5 |
| *ITGA4*** | 0.6 | 1.0 | 0.8 | 0.6 | 4.1* | 1.2 | 1.2 | 0.8 | 4.3* | 0.5 |
| *ITGA5* | 0.1 | -1.1 | 0.2 | -0.9 | -0.5 | 0.5 | 2.0 | -0.1 | -0.5 | 1.2 |
| *ITGA7*** | 1.9 | 1.5 | 2.5 | 1.9 | 1.6 | 2.5 | 1.2 | 2.5 | 3.7 | 2.5 |
| *ITGA8*** | 0.6 | 0.5 | 0.4 | 2.5 | 0.8 | 2.5 | 1.2 | 0.8 | 0.6 | 1.5 |
| *ITGA9*** | 1.2 | 1.0 | 0.4 | 0.6 | 1.6 | 1.2 | 2.5 | 0.8 | 0.6 | 1.5 |
| *ITGB1* | 2.6 | 0.9 | 1.8 | 1.1 | 1.8 | 0.5 | 0.5 | -0.1 | 0.5 | 1.2 |
| *ITGB3* | 1.7 | 0.5 | -1.3 | 1.1 | -0.5 | 0.5 | 1.5 | 0.9 | -0.5 | -1.1 |
