# Supplementary material for: Digital spatial profiling of segmental outflow regions in trabecular meshwork reveals a role for ADAM15
Source: PLoS One. 2024 Feb 23;19(2):e0298802. doi: 10.1371/journal.pone.0298802 (PMC10889904; doi:10.1371/journal.pone.0298802)
Supplement: S1 File — (PDF) [file pone.0298802.s004.pdf]

OREGON HEALTH & SCIENCE UNIVERSITY

IRB MEMO

Research Integrity Office
3181 SW Sam Jackson Park Road - L106RI
Portland, OR 97239-3098
(503)494-7887 irb@ohsu.edu

NOT HUMAN RESEARCH

January 25, 2023

Dear Investigator:

On 1/25/2023, the IRB reviewed the following submission:

Title of Study:	Thrombospondin-1 in normal and glaucomatous trabecular meshwork
Investigator:	Kate Keller
IRB ID:	STUDY00025374
Funding:	Name: DHHS NIH Natl Eye Inst, PPQ #: 1020808, Funding Source: EY032590

The IRB determined that the proposed activity is not research involving human subjects. IRB review and approval is not required.

Certain changes to the research plan may affect this determination. Contact the IRB Office if your project changes and you have questions regarding the need for IRB oversight.

If this project involves the collection, use, or disclosure of Protected Health Information (PHI), you must comply with all applicable requirements under HIPAA. See the HIPAA and Research website and the Information Privacy and Security website for more information.

Sincerely,

The OHSU IRB Office
